# Effects of heat stimulation after high-intensity interval training on serum adipokines, eHSP72, and immunoglobulins in men with obesity

**DOI:** 10.1016/j.jesf.2025.11.002

**Published:** 2025-11-07

**Authors:** Do Hyun Kim, Eun Sook Kim, Jin Seok Lee, Sung Jin Yoon

**Affiliations:** a Department of Physical Education, Korea University, Seoul, Republic of Korea; b Department of Physical Education, Daegu National University of Education, Daegu, Republic of Korea

**Keywords:** Heat stimulation, High-intensity interval training, Adipokine, eHSP72, Immunoglobulin, Obesity

## Abstract

**Background/objectives:**

Excessive body fat causes imbalances in adipokines and impairs immune function, and not only exercise but also heat stimulation can promote fat reduction and induce extracellular heat shock protein 72 (eHSP72) expression, thereby improving immune function. Therefore, we examined the effects of heat stimulation after HIIT on adipokines, eHSP72, and immunoglobulin levels in men with obesity.

**Methods:**

Forty-eight men were randomly assigned to one of the following three groups: high-intensity interval training followed by heat stimulation (HIITHS), moderate-intensity continuous training followed by heat stimulation (MICTHS), and heat stimulation (HS). The 12-week intervention was performed three times per week. Body composition and blood marker levels were assessed before and after the intervention.

**Results:**

The HIITHS and MICTHS groups showed significant improvements in body weight, body mass index (BMI), and body fat percentage. Adiponectin levels increased, whereas leptin levels decreased in all groups. eHSP72 and immune marker levels (IgA, IgG) increased, with the greatest eHSP72 response observed in the HIITHS group. The IgE levels decreased in the HIITHS and HS groups.

**Conclusion:**

These results suggest that combining HIIT with heat stimulation improves serum adipokines, eHSP72, and immunoglobulins in men with obesity, indicating that this approach may serve as a safe and effective intervention.

## 1 Introduction

The global prevalence of obesity has been consistently increasing over the past four decades. As of 2022, approximately 16 % of the world's adult population, equivalent to one in every eight individuals, was classified as obese.^1^ Obesity is a major risk factor for various chronic diseases, including type 2 diabetes, hypertension, cardiovascular disease, and dyslipidemia.^2^^,^^3^ While obesity is caused by various factors, reduced physical activity levels in modern society are considered the primary contributor.^4^ Therefore, obesity is a serious societal issue, and effective methods for its prevention and management are required.

Obesity is characterized by the excessive accumulation of adipose tissue, which leads to adipose tissue dysfunction and an imbalances in the adipokine concentrations.^5^^,^^6^ Adipokines are adipose-derived proteins with endocrine and metabolic functions, among which adiponectin and leptin are the most prominent. Abnormal adipokine fluctuations increase the expression of inflammatory cytokines.^7^, ^8^, ^9^ Sustained elevated levels of these inflammatory cytokines can result in chronic inflammation, which can compromise immune function by delaying responses to viral pathogens, diminishing bactericidal capacity, and reducing lymphocyte levels.^10^, ^11^, ^12^ As excessive body fat leads to adipokine imbalances and impaired immune function, improvements in body composition must be prioritized for the management of obesity.

Physical activity is a recognized approach to optimizing body composition in individuals with obesity, and moderate-intensity aerobic training is known to effectively improve body composition, lipid metabolism, inflammatory responses, and adipokine profiles.^13^, ^14^, ^15^, ^16^ High-intensity interval training (HIIT) involves a shorter exercise duration than does moderate-intensity aerobic training, and has emerged as a practical alternative for individuals with limited time.^17^ Even when performed for relatively shorter durations than moderate-intensity aerobic training, high-intensity interval training can improve adipokine profiles and immune function.^18^, ^19^, ^20^, ^21^ Despite its time-efficient advantage, HIIT is associated with the "J-shaped relationship theory," according to which immune function may be suppressed for approximately 3–72 h after exercise, thereby increasing susceptibility to upper respiratory tract infections. This effect can be exacerbated in individuals with obesity and low fitness levels.^22^^,^^23^ Therefore, practical methods should be developed that can be safely applied to individuals with obesity.

In addition to exercise interventions, approaches such as dietary strategies and environmental modifications have been explored for weight reduction and immune enhancement, and recent studies have reported that heat exposure, which induces an increase in body temperature, may further contribute to reductions in body fat.^24^, ^25^, ^26^, ^27^ An increase in body temperature induced by heat stimuli activates the sympathetic nervous system, thereby promoting lipid metabolism and energy expenditure.^28^^,^^29^ Moreover, heat exposure results in vasodilation and increased blood flow, which enhances circulation. This facilitates the delivery of oxygen, nutrients, white blood cells, and antibodies, which exerts beneficial effects on tissue regeneration and growth.^30^^,^^31^ Notably, heat exposure induces the expression of extracellular heat shock protein 72 (eHSP72), a cytoprotective protein that assists in the repair of damaged proteins involved in inflammatory and metabolic processes.^32^, ^33^, ^34^, ^35^ eHSP72 is released into the bloodstream in response to physiological stress such as heat exposure or exercise, and is considered a useful biomarker associated with immune regulation and systemic inflammation.^36^^,^^37^ Furthermore, eHSP72 plays a pivotal role in regulating adipokine balance and glucose metabolism.^38^ For eHSP72 expression to be effectively stimulated, core body temperature must rise by approximately 1 °C–4 °C above baseline levels.^39^, ^40^, ^41^ However, the ability to achieve such an elevation in body temperature solely through exercise is often limited. Therefore, we hypothesized that heat exposure after exercise may serve as an effective means of stimulating eHSP72 expression, thereby enhancing adipokine regulation and immune function.

Furthermore, extracellular HSP72 (eHSP72), which is induced by both exercise and heat exposure, may play a crucial role in modulating immune function. Once released into the bloodstream, eHSP72 can function as a danger-associated molecular pattern (DAMP) that interacts with toll-like receptors (TLRs) on immune cells, thereby activating intracellular signaling pathways such as nuclear factor kappa B (NF-κB) and mitogen-activated protein kinase (MAPK).^42^^,^^43^ These pathways regulate the production of pro-inflammatory and anti-inflammatory cytokines, influence B cell activation, and ultimately contribute to immunoglobulin synthesis (IgA, IgG, IgM).^44^^,^^45^ Through these mechanisms, eHSP72 may mediate the immunomodulatory effects observed following heat stimulation after high-intensity interval training. Therefore, understanding the underlying molecular pathways by which HSP72 influences immune responses may provide valuable insights for developing combined exercise and heat-based interventions targeting both metabolic and immune health in individuals with obesity.

To this end, we herein investigated the use of heat exposure after high-intensity interval training to enhance eHSP72 expression and support immune function in individuals with obesity. Through this approach, we sought to determine whether this combined intervention can be safely and effectively applied to individuals with obesity. Although numerous researchers have investigated the independent effects of exercise and heat exposure, studies exploring their combined effect remains limited. Therefore, the objective of this study was to examine the effects of heat stimulation after high-intensity interval training on adipokines, eHSP72, and immunoglobulins levels in men with obesity, and to provide foundational evidence for the development of effective exercise and heat-based interventions.

## 2 Materials and methods

### 2.1 Participants

**Fig. 1:**
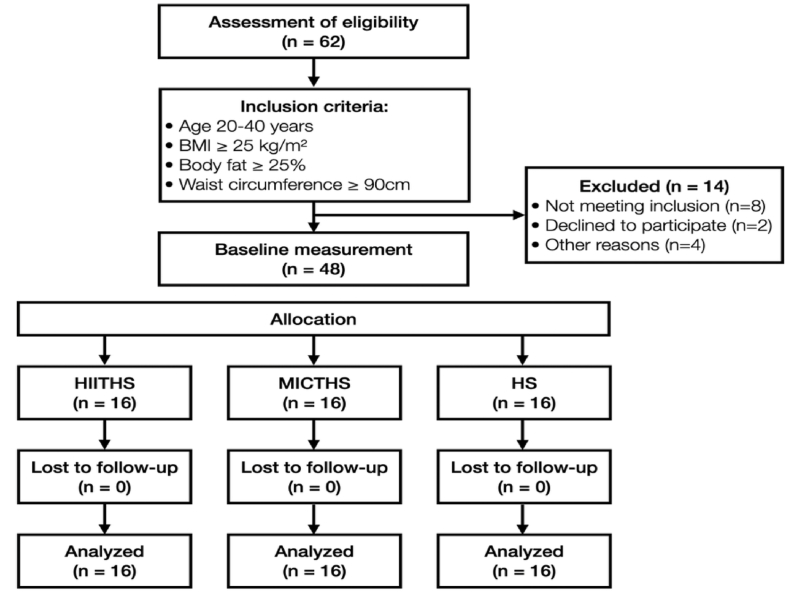
CONSORT flow diagram. HIITHS: high-intensity interval training followed by heat stimulation, MICTHS: moderate-intensity continuous training followed by heat stimulation, HS: heat stimulation.

This study was conducted among adult men with obesity aged 20–40 years. Participants met the following obesity criteria: BMI ≥25 kg/m^2^, body fat percentage ≥25 %, and waist circumference ≥90 cm.^46^ Participants were excluded if they had any history of cardiovascular, metabolic, musculoskeletal, or endocrine disorders; were taking medications that could affect metabolism; had participated in regular exercise programs within the previous 3 months; or had prior exposure to heated environments. The sample size was calculated using G Power software version 3.1 (Universität Düsseldorf, Germany). For a repeated-measures ANOVA with a within-between interaction, assuming an effect size (η^2^) of .06, a significance level (α) of .05, and a statistical power of .85, the required sample size was determined to be 48 participants. The assumed medium effect size (η^2^ = .06) was based on previous intervention studies examining the effects of high-intensity interval training and/or heat exposure on adipokines and immune markers in individuals with obesity. Prior research reported partial eta-squared values ranging from .05 to .10 for similar outcome measures.^19^, ^20^, ^21^ Given the novelty of combining high-intensity interval training with post-exercise heat stimulation, we adopted a conservative medium effect size according to Cohen's classification to ensure adequate statistical power.^47^Accordingly, 48 participants were recruited, and their baseline characteristics are presented in Table 1 and the study flow diagram presented in Fig. 1. Thisstudy was conducted with the approval of the Korea University Bioethics Committee(KUIRB-2024-0134-01). In accordance with the Declaration of Helsinki, the purpose and procedures of this study were fully explained to all participants, who voluntarily provided written informed consent prior to enrollment.

**Table 1:** Baseline characteristics of the participants.

Variable	HITHS(n = 16)	MICTHS(n = 16)	HS(n = 16)	F	P value
Age (yrs)	27.68 ± 4.51	25.06 ± 5.11	28.12 ± 4.68	1.92	.158
Height (cm)	174.65 ± 6.54	175.30 ± 4.13	174.47 ± 4.59	.112	.894
Weight (kg)	87.79 ± 5.79	88.51 ± 6.10	88.20 ± 5.77	.063	.939
BMI (kg/m^2^)	28.89 ± 2.84	28.85 ± 2.49	29.03 ± 2.05	.024	.976
Body fat percentage (%)	31.75 ± 3.56	31.31 ± 3.40	32.06 ± 2.79	.909	.410
Waist circumference (cm)	100.43 ± 5.48	101.50 ± 4.89	102.62 ± 8.30	.467	.630

Values are presented as means ± SD. HIITHS: high-intensity interval training followed by heat stimulation, MICTHS: moderate-intensity continuous training followed by heat stimulation, HS: heat stimulation, BMI: body mass index.

### 2.2 Study design

**Fig. 2:**
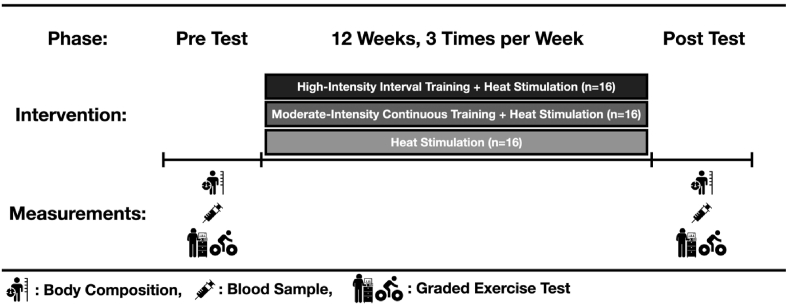
Study design.

This intervention was performed three times per week for 12 weeks. Participants were randomly assigned to one of three groups: (1) high-intensity interval training followed by heat stimulation (HIITHS), (2) moderate-intensity aerobic training followed by heat stimulation (MICTHS), and (3) heat stimulation only (HS). Randomization was conducted using a computer-generated randomization sequence created in IBM SPSS version 26.0 (IBM Corp., Armonk, NY) after baseline measurements were completed. This procedure was implemented to ensure group equivalence at baseline and to minimize selection bias, in accordance with recommendations from previous intervention trials.^48^^,^^49^ The inclusion of these three groups allowed us to compare the effects of exercise intensity combined with heat stimulation and to isolate the independent contribution of heat stimulation alone, thereby providing a comprehensive evaluation of the combined and separate effects of exercise and heat exposure on metabolic and immune outcomes. To minimize potential confounding factors, participants were instructed to maintain their habitual dietary patterns and lifestyle throughout the 12-week intervention, and they were verbally reminded at each session not to alter their regular eating habits. The study design is summarized in Fig. 2.

### 2.3 Measurement

(1) Body composition

Body composition was measured at both baseline and after the intervention. The participants were instructed to abstain from eating or exercising for at least 12 h before measurement. While wearing only a short-sleeved cotton shirt and cotton shorts, their height, weight, body fat percentage, and BMI were assessed using an automatic height-weight scale (BSM 340, *Biospace*, Korea) and a bioelectrical impedance analyzer (Inbody 570, *Biospace*, Korea). Waist circumference was measured with the participants standing in a relaxed position. A measuring tape was wrapped horizontally around the midpoint between the lowest rib and the iliac crest, and the measurement was recorded immediately after exhalation.

(2) Blood Sampling

After a 12-h fast and in a resting state, blood samples were collected from the antecubital vein of all the participants at baseline and at least 48 h after the final exercise session. According to previous studies, extracellular heat shock proteins and inflammatory cytokines generally return to baseline levels within 24–48 h after exercise. Based on this evidence, blood sampling in the present study was performed at least 48 h after the final exercise session to minimize the influence of acute responses and to more accurately reflect the chronic adaptations induced by the 12-week intervention.^36^^,^^50^ The blood was allowed to clot at room temperature and then centrifuged at 3000 rpm for 10 min at 4 °C to separate the serum. All serum samples were aliquoted and stored at −80 °C until analysis. Serum concentrations of eHSP72, adiponectin, leptin, and immunoglobulins (IgA, IgG, IgM, IgD, IgE) were measured using enzyme-linked immunosorbent assay (ELISA) kits, which are widely used in previous studies. eHSP72 levels were determined using a Human HSP72 ELISA kit (Enzo Life Sciences, USA), which is validated for serum and plasma samples and commonly used in exercise physiology and heat stress studies. Adiponectin and leptin concentrations were assessed using Quantikine® ELISA kits (R&D Systems, Minneapolis, MN, USA), which are highly specific and sensitive for human serum samples. Immunoglobulin A (IgA), IgG, and IgM levels were analyzed using a Human Immunoglobulin ELISA Kit (Abcam, Cambridge, UK), following the manufacturer's protocol. All assays were performed in duplicate, and the optical density was measured at 450 nm using a microplate reader (e.g., BioTek ELx808, Winooski, VT, USA). The intra-assay and inter-assay coefficients of variation (CV) for all biomarkers were maintained below 10 %. All serum samples were coded and analyzed in a blinded manner by the investigator, and statistical analyses were performed using coded datasets to minimize potential bias.

(3) Graded exercise test

Graded exercise tests were conducted at baseline and after the intervention. The participants were instructed to refrain from excessive physical activity for 24 h before the test and to arrive at the laboratory at least 1 h in advance to ensure adequate rest. Following a light stretching warm-up, the participants wore a gas mask and rested for approximately 5 min before the test began.

The test was performed using a cycle ergometer (Powermax II, *Combi*, Japan) based on the Astrand protocol. Maximal oxygen uptake (VO_2_max) was measured using a gas analyzer (Quark b2, *Cosmed*, Italy), and heart rate was continuously monitored using a wireless heart rate monitor (RS 400, *Polar*, Finland). According to the Astrand protocol, the initial workload was set at 300 kg m/min (50 W) and was increased by 150 kg m/min (25 W) every 2 min until volitional exhaustion. The test was terminated when the respiratory exchange ratio (RER) exceeded 1.15, or when the oxygen uptake plateaued despite an increase in workload. During the test, a metronome was set at 100 beats per minute to help the participants maintain a pedaling cadence of 50 rpm.

### 2.4 Intervention protocol

The HIITHS group performed high-intensity interval training followed by heat stimulation; the MICTHS group performed moderate-intensity aerobic training followed by heat stimulation, and the HS group underwent heat stimulation only. Both high-intensity interval training and moderate-intensity aerobic training were performed using a cycle ergometer (MK-2100H, *Melkin*, Korea). Each session began with a 5-min cycling warm-up at 40–50 % HRmax, followed by stretching.

High-intensity interval training comprised of four bouts, each lasting 4 min at 85–95 % HRmax, with 3-min active recovery periods at 70 % HRmax between bouts. To ensure that participants reached target HR levels during each high-intensity interval, they were instructed to increase cycling speed and resistance at the start of each work bout so that HR reached 85–90 % HRmax within the first 30–60 s of each interval. Moderate-intensity aerobic training involved 40 min of cycling at 60–70 % HRmax. The participants' heart rates were monitored to throughout the sessions to ensure that the target intensity was maintained, and verbal encouragement was provided to promote consistent pedaling. All participants successfully adhered to the training protocol without any missed sessions or protocol violations throughout the 12-week intervention period.

Heat stimulation was administered after the training and consisted of 20 min of hot water immersion in a foldable bathtub (Foldable Bathtub Oval, *4onemillion*, China). The participants wore identical swimsuits and were immersed in a seated position with water up to the clavicle. The water temperature was maintained at 40 °C using a water heater (WAT-730B, *Ystage*, Korea).^51^^,^^52^ Water (TES 1300, *TES*, Taiwan), tympanic (IRT-6520, *Braun*, USA), and sublingual temperature (Dt-R1221AWG, *iProven*, USA) were continuously monitored every 5 min to assess the participants’ physiological responses ensure safety throughout the heat exposure sessions.

To improve accessibility and practicality, portable foldable bathtubs and standard commercial water heaters were used, allowing for easy setup and reproducibility in various settings. Participants were screened for contraindications such as cardiovascular disease, impaired thermoregulation, or other medical conditions that could increase the risk of heat-related adverse events. Verbal communication was maintained throughout each session to monitor participants’ comfort and tolerance, and sessions were discontinued immediately if any signs of heat intolerance or discomfort appeared. These safety measures were implemented to ensure participant adherence and minimize potential risks associated with heat exposure.

### 2.5 Data analysis

All statistical analyses were performed using IBM SPSS version 26.0 (IBM Corp., Armonk, NY). Data are presented as mean ± standard deviation (SD). To examine the changes in men with obesity according to group and time, a two-way repeated measures ANOVA was conducted. When a significant interaction effect was found, Bonferroni post hoc tests were used to compare the differences between groups or time points. If no interaction effects were observed, the main effect of each independent variable was analyzed. The assumption of normality was assessed using the Shapiro–Wilk test. The assumption of sphericity was evaluated using Mauchly's test of sphericity. When sphericity was violated, the Greenhouse–Geisser correction was applied. Statistical significance was set at α = .05.

## 3 Results

### 3.1 Changes in body temperature

The results of the tympanic temperature-change analysis are presented in Table 2. A significant interaction effect was found between measurement time and group (*F*(2, 45) = 47.193, *p* = .001, *η*_*p*_^*2*^ = .677). The post hoc analysis revealed a significant increase in tympanic temperature from baseline to after the intervention in the HIITHS (Δ% = 3.70, 95 % CI [1.58, 5.82], *d* = 5.29) and MICTHS (Δ% = 3.48, 95 % CI [1.69, 5.27], *d* = 5.88) groups (*p* < .05). Upon comparing the groups, the participants’ tympanic temperature after the intervention were significantly higher in the HIITHS and MICTHS groups than in the HS group (*p* < .05).

**Table 2:** Changes in body temperature after first session of intervention.

Variable	Group	Pre	Post Treatment	Δ%(95 %CI)		*F*	*P*(*ηp*^2^)
Tympanic temperature (°C)	HIITHS	36.46 ± .28	37.81 ± .23^#a^	3.70(1.58,5.82)	Time	1133.594	.001∗(.962)
	MICTHS	36.48 ± .19	37.75 ± .24^#a^	3.48(1.69,5.27)	Group	9.232	.001∗(.291)
	HS	36.53 ± .23	37.17 ± .21	1.75(-.07,3.57)	Time × Group	47.193	.001∗(.677)
Sublingual temperature (°C)	HIITHS	36.38 ± .19	37.78 ± .19^#a^	3.85(2.28,5.42)	Time	582.899	.001∗(.928)
	MICTHS	36.41 ± .23	37.78 ± .27^#a^	3.76(1.68,5.84)	Group	11.042	.001∗(.329)
	HS	36.46 ± .18	37.16 ± .37	1.921(-.48,4.32)	Time × Group	21.983	.001∗(.494)

Values are presented as means ± SD. ∗^:^*p* < .05, ^#^: significantly different from pre, ^a^: significantly different from HS. HIITHS: high-intensity interval training followed by heat stimulation, MICTHS: moderate-intensity continuous training followed by heat stimulation.

Table 2 presents the results of the sublingual temperature-change analysis. There was a significant interaction effect between the measurement time and group (*F*(2, 45) = 21.983, *p* = .001, *η*_*p*_^*2*^ = .494). The post hoc analysis revealed a significant increase in sublingual from baseline to after the intervention in the HIITHS (Δ% = 3.85, 95 % CI [2.28, 5.42], *d* = 4.42) and MICTHS (Δ% = 3.76, 95 % CI [1.68, 5.84], *d* = 4.02) groups (*p* < .05). Upon comparing the groups, the participants’ tympanic temperature after the intervention were significantly higher in the HIITHS and MICTHS groups than in the HS group (*p* < .05).

### 3.2 Changes in body composition

The results of the body weight-change analysis are presented in Table 3. A significant interaction effect was found between measurement time and group (*F*(2, 45) = 11.814, *p* = .0, *η*_*p*_^*2*^ = .344). The post hoc analysis revealed a significant decrease in weight from baseline to after the intervention in the HIITHS (Δ% = −4.92, 95 % CI [−6.56, −3.28], *d* = 1.60) and MICTHS (Δ% = −4.97, 95 % CI [−6.56, −4.28], *d* = 3.83) groups (*p* < .05). Upon comparing the groups, the participants’ weights after the intervention were significantly lower in the HIITHS and MICTHS groups than in the HS group (*p* < .05).

**Table 3:** Changes in body composition after 12 weeks of intervention.

Variable	Group	Pre	Post	Δ%(95 %CI)	Cohen's *d*		*F*	*P*(*ηp*^2^)
Body Weight (kg)	HIITHS	87.79 ± 5.79	83.47 ± 6.18^#a^	−4.92(-6.64, −2.00)	1.05	Time	24.530	.001∗(.353)
	MICTHS	88.51 ± 6.10	84.11 ± 6.21^#a^	−4.97(-6.91, −1.89)	.94	Group	.824	.445(.035)
	HS	88.20 ± 5.77	88.01 ± 6.71	−.22(-2.17, 1.75)	.08	Time × Group	4.971	.011∗(.181)
BMI (kg/m^2^)	HIITHS	28.89 ± 2.84	27.45 ± 2.63^#^	−4.91(-6.64, −3.32)	.94	Time	24.695	.001∗(.354)
	MICTHS	28.85 ± 2.49	27.38 ± 1.97^#^	−5.10(-5.62, −4.30)	.90	Group	.720	.492(.031)
	HS	29.03 ± 2.05	28.93 ± 2.29	−.34(-5.71, −1.27)	.09	Time × Group	4.990	.011∗(.182)
Body fat percentage (%)	HIITHS	31.75 ± 3.56	25.81 ± 4.46^#a^	−18.7(-25.5,-11.9)	.89	Time	33.173	.001∗(.424)
	MICTHS	31.31 ± 3.40	27.43 ± 5.09^#a^	−12.4(-21.5,-3.2)	.72	Group	2.661	.081(.106)
	HS	32.06 ± 2.79	30.43 ± 3.66	−5.1(-12.0,1.9)	.39	Time × Group	3.539	.037∗(.136)

Values are presented as means ± SD. ∗^:^*p* < .05, ^#^: significantly different from pre, ^a^: significantly different from HS. HIITHS: high-intensity interval training followed by heat stimulation, MICTHS: moderate-intensity continuous training followed by heat stimulation, HS: heat stimulation, BMI: body mass index.

Table 3 presents the results of the BMI-change analysis. There was a significant interaction effect between the measurement time and group (*F*(2, 45) = 4.990, *p* = .011, *η*_*p*_^*2*^ = .182). The post hoc analysis revealed a significant decrease in BMI from baseline to after the intervention in the HIITHS (Δ% = −4.91, 95 % CI [−6.44, −3.32], *d* = .94) and MICTHS (Δ% = −5.10, 95 % CI [−5.62, −4.30], *d* = .90) groups (*p* < .05). However, no significant differences were found between the groups (*p* > .05).

In the body fat percentage-change analysis (Table 3), we found a significant interaction effect between the measurement time and group (*F*(2, 45) = 3.539, *p* = .037, *η*_*p*_^*2*^ = .136). The post hoc analysis revealed a significant decrease in body fat percentage from baseline to after the intervention in the HIITHS (Δ% = −18.7, 95 % CI [−25.5, −11.9], *d* = 1.46) and MICTHS (Δ% = −12.4, 95 % CI [−21.5, −3.2], *d* = .72) groups (*p* < .05). Upon comparing the groups, the participants’ body fat percentages after the intervention were significantly lower in the HIITHS and MICTHS groups than in the HS group (*p* < .05).

### 3.3 Changes in adipokines levels

**Fig. 3:**
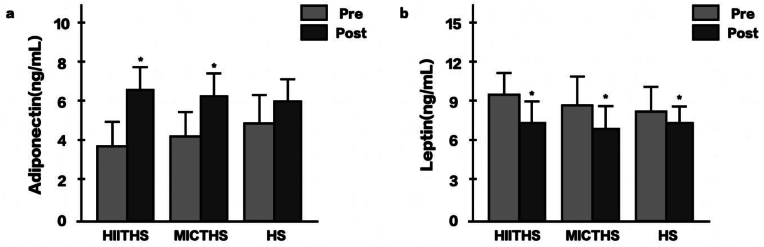
Changes in serum adipokines levels after 12 weeks of intervention. *p* < .05, ∗: significantly different from pre. HIITHS: high-intensity interval training followed by heat stimulation, MICTHS: moderate-intensity continuous training followed by heat stimulation, HS: heat stimulation.

Fig. 3a show the results of the adiponectin-change analysis. There was a significant interaction effect between the measurement time and group (*F*(2, 45) = 24.530, *p* = .011, *η*_*p*_^*2*^ = .181). The post hoc analysis revealed a significant increase in adiponectin level from baseline to after the intervention in the HIITHS (Δ% = 4.92, 95 % CI [−6.64, −2.00], *d* = 1.05) and MICTHS (Δ% = 4.97, 95 % CI [−6.91, −1.89], *d* = .94) groups (*p* < .05). However, no significant differences were found between the groups (*p* > .05). In the leptin-change analysis (Fig. 3b), no significant interaction effect was found between the measurement time and group (*F*(2, 45) = 1.713, *p* = .192, *η*_*p*_^2^ = .071). The main-effect analysis revealed a significant decrease in leptin level from baseline to after the intervention in all three groups: HIITHS (Δ% = −25.42, 95 % CI [−34.58, −16.26], *d* = 1.09), MICTHS (Δ% = −24.69, 95 % CI [−33.82, −15.56], *d* = 1.04), and HS (Δ% = −20.92, 95 % CI [−29.89, −11.95], *d* = .86) groups (*p* < .05). However, no significant differences were found between the groups (*p* > .05).

### 3.4 Changes in eHSP72 levels

**Fig. 4:**
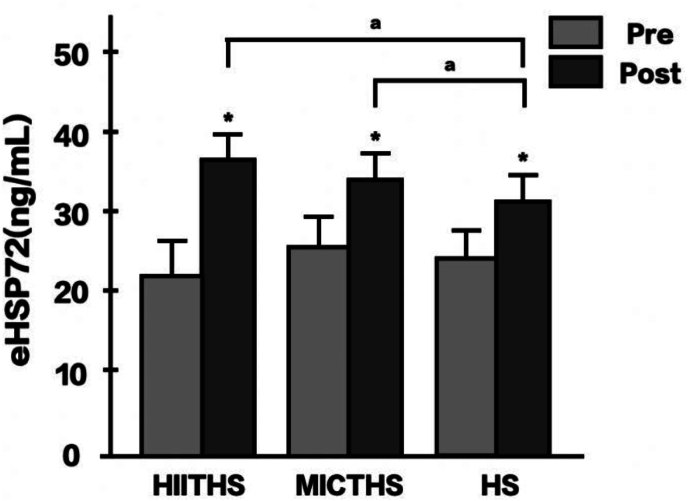
Changes in serum eHSP72 levels after 12 weeks of intervention. HIITHS: high-intensity interval training followed by heat stimulation, MICTHS: moderate-intensity continuous training followed by heat stimulation, HS: heat stimulation, eHSP72: extracellular heat shock protein 72. *p* < .05, ∗: significantly different from pre, ^a^: significantly different from HS.

Fig. 4 shows the results of the eHSP72-change analysis. A significant interaction effect was found between the measurement time and group (*F*(2, 45) = 8.168, *p* = .001, *η*_*p*_^2^ = .266). The post hoc analysis revealed a significant increase in eHSP72 level from baseline to after the intervention in all three groups: HIITHS (Δ% = 59.91, 95 % CI [48.09, 71.73], *d* = 3.61), MICTHS (Δ% = 38.00, 95 % CI [26.65, 49.35], *d* = 2.40), and HS (Δ% = 28.67, 95 % CI [17.60, 39.74], *d* = 1.49) groups (*p* < .05). Upon comparing the groups, the eHSP72 levels after the intervention were significantly higher in the HIITHS and MICTHS groups than in the HS group (*p* < .05).

### 3.5 Changes in immunoglobulin levels

In the IgA-change analysis (Table 4), we found no significant interaction effect between the measurement time and group (*F*(2, 34) = .234, *p* = .793, *ηp*^2^ = .010). The main effect analysis revealed a significant increase in IgA level from baseline to after the intervention in all three groups: HIITHS (Δ% = 22.25, 95 % CI [8.57, 35.91], *d* = .87), MICTHS (Δ% = 17.57, 95 % CI [7.28, 27.84], *d* = .91), and HS (Δ% = 19.33, 95 % CI [9.29, 29.27], *d* = 1.04) groups (*p* < .05). However, no significant differences were found between the groups (*p* > .05).

**Table 4:** Changes in serum immunoglobulin levels after 12 weeks of intervention.

Variable	Group	Pre	Post	Δ%(95 %CI)	Cohen's *d*		*F*	*P*(*ηp*^2^)
**IgA (mg/dL)**	HIITHS	173.06 ± 43.19	211.56 ± 36.26^#^	22.25(8.57,35.91)	.77	Time	40.587	.001∗(.474)
	MICTHS	168.37 ± 32.87	197.93 ± 46.23^#^	17.57(7.28,27.84)	.81	Group	.378	.688(.017)
	HS	175.56 ± 37.82	209.50 ± 38.73^#^	19.33(9.39,29.27)	.94	Time × Group	.234	.793(.010)
**IgG (mg/dL)**	HIITHS	1062.87 ± 131.39	1268.75 ± 126.87^#^	18.71(9.62,27.78)	.78	Time	64.977	.001∗(.591)
	MICTHS	1016.81 ± 109.98	1198.18 ± 128.88^#^	17.83(9.78,25.89)	.71	Group	1.670	.200(.069)
	HS	994.37 ± 144.14	1237.62 ± 152.11^#^	24.47(13.87,35.05)	.85	Time × Group	.476	.624(.021)
**IgM (mg/dL)**	HIITHS	121.31 ± 22.72	132.50 ± 22.75	9.22(4.18,14.27)	.98	Time	10.752	.002∗(.193)
	MICTHS	124.31 ± 24.85	129.56 ± 28.22	4.22(.30,8.15)	.57	Group	.358	.701(.016)
	HS	119.06 ± 26.87	122.50 ± 22.27	2.89(-5.76,11.53)	.18	Time × Group	1.342	.272(.056)
**IgD (mg/dL)**	HIITHS	2.59 ± 1.34	3.24 ± 1.90	53.49(20.85,86.16)	.87	Time	19.292	.001∗(.300)
	MICTHS	2.23 ± .90	2.54 ± 1.08	25.23(4.50,45.92)	.65	Group	.630	.537(.027)
	HS	2.62 ± 1.70	3.01 ± 1.63	32.39(-5.62,70.33)	.45	Time × Group	.994	.378(.042)
**IgE (mg/dL)**	HIITHS	106.85 ± 16.07	93.91 ± 14.89^#^	−11.91(-20.46,-3.37)	.74	Time	16.645	.001∗(.270)
	MICTHS	97.73 ± 16.35	92.55 ± 13.09	−5.94(-15.34,-3.46)	.34	Group	.976	.380(.042)
	HS	106.27 ± 13.72	94.54 ± 14.56^#^	−11.04(-19.04,-3.04)	.73	Time × Group	.989	.385(.042)

Values are presented as mean ± SD. ∗: *p* < .05, ^#^: significantly different from pre. HIITHS: high-intensity interval training followed by heat stimulation, MICTHS: moderate-intensity continuous training followed by heat stimulation, HS: heat stimulation, IgA: immunoglobulin A, IgG: immunoglobulin G, IgM: immunoglobulin M, IgD: immunoglobulin D, IgE: immunoglobulin E.

Table 4 show the results of the IgG-change analysis. No significant interaction effect was found between the measurement time and group (*F*(2, 45) = .476, *p* = .624, *η*_*p*_^2^ = .021). The main-effect analysis revealed a significant increase in IgG level from basement to after the intervention in all three groups: HIITHS (Δ% = 18.71, 95 % CI [6.22, 27.78], *d* = 1.10), MICTHS (Δ% = 17.83, 95 % CI [9.78, 25.89], *d* = 1.18), and HS (Δ% = 24.47, 95 % CI [13.87, 35.05], *d* = 1.23) groups (*p* < .05). However, no significant differences were found between the groups (*p* > .05).

The results of the IgM-change analysis are presented in Table 4. No significant interaction effect was found between the measurement time and group (*F*(2, 45) = 1.342, *p* = .272, *η*_*p*_^2^ = .056). The main-effect analysis revealed no significant differences between the measurement times or groups (*p* > .05).

The results of the IgD change analysis are presented in Table 4. No significant interaction effect was found between the measurement time and group (*F*(2, 45) = .994, *p* = .378, *η*_*p*_^2^ = .042). The main effect analysis revealed no significant differences between the measurement times or groups (*p* > .05).

Table 4 show the results of the IgE-change analysis. No significant interaction effect was found between the measurement time and group (*F*(2, 45) = .989, *p* = .385, *η*_*p*_^2^ = .042). The main-effect analysis revealed a significant decrease in IgE level from baseline to after the intervention in the HIITHS (Δ% = −11.91, 95 % CI [−20.46, −3.37], *d* = .74) and HS (Δ% = −13.04, 95 % CI [−19.04, −3.04], *d* = .73) groups (*p* < .05). However, no significant differences were found between the groups (*p* > .05).

### 3.6 Safety outcomes

No adverse events, heat-related complications, or safety concerns were observed during the 12-week intervention period. All 48 participants (16 per group) completed the protocol without missed sessions or early terminations related to heat intolerance.

No participants reported symptoms commonly associated with heat stress, including dizziness, nausea, vomiting, excessive fatigue, headache, palpitations, chest discomfort, shortness of breath, muscle cramps, or feelings of faintness.

Mean post-heat exposure tympanic temperature was 37.78 ± .24 °C (range: 37.16–38.10 °C), and mean sublingual temperature was 37.78 ± .26 °C (range: 37.16–38.30 °C). All measurements remained within normal physiological limits, and no sessions required early termination or temperature management interventions.

These findings indicate that 20-min hot water immersion at 40 °C was well-tolerated and safe in men with obesity.

## 4 Discussion

In several studies, researchers have investigated the prevention and treatment of obesity. In addition to exercise-based interventions, recent findings have suggested that heat stimulation may induce additional physiological stress responses and potentially enhance the overall effects of exercise. However, despite the individual effects of exercise and heat stimulation having been studied, the combined effect of post-exercise heat stimulation has not been thoroughly investigated. Therefore, we aimed to examine the effects of heat stimulation after high-intensity interval training on adipokines, eHSP72, and immunoglobulins levels in men with obesity.

Obesity is characterized by the excessive accumulation of adipose tissue, which disrupts adipokine homeostasis. Adipokines secreted by adipose tissue are hormones involved in various metabolic processes including energy metabolism, appetite regulation, and insulin sensitivity.^53^^,^^54^ Among these, adiponectin promotes glucose metabolism and fatty acid oxidation. Herein, adiponectin levels significantly increased in both the HIITHS and MICTHS groups. Despite the short duration of each session, high-intensity interval training increases energy expenditure and depletes muscle glycogen, thereby activating the AMPK and PGC-1α pathways that enhance mitochondrial biogenesis and fat oxidation.^55^, ^56^, ^57^ Rocha-Rodrigues et al.^58^ have reported a positive correlation between a reduction in body fat percentage and an increase in adiponectin levels. Accordingly, after 12 weeks of training, both the HIITHS and MICTHS groups in our study showed significant reductions in body weight, BMI, and body fat percentage, which likely contributed to the observed increase in adiponectin levels.

Leptin, which is involved in appetite regulation and energy metabolism, was significantly decreased in all three groups. Koçak et al.^59^ reported a decrease in leptin level after 15 sessions of heat stimulation in obese adults, suggesting that heat stimulation induced microvascular dilation promotes blood circulation in adipose tissue, which is associated with decreased leptin levels. Similarly, we found a decrease in leptin levels in the group that received heat stimulation for 12 weeks. Therefore, the combined intervention of 12 weeks of high-intensity interval training and heat stimulation improved the body composition and adipokine profiles of our participants, which confirms its potential as a method for reducing obesity.

Following the 12-week training intervention, eHSP72 levels significantly increased in all three groups. eHSP72 is known as a protein that activates cellular protective mechanisms in stress situations; external stress and an elevated body temperature activate heat shock factor 1 (HSF 1), which promotes eHSP72 expression.^60^^,^^61^ In this study, both the HIITHS and MICTHS groups received heat stimulation after exercise, and their eHSP72 levels were significantly higher than those in the group that received heat stimulation alone. In post-exercise heat stimulation, the mechanical stress from exercise is combined with an increased body temperature from heat stimulation, which potentially enhances cellular stress responses.^62^, ^63^, ^64^ In our study, the correlation between heat exposure and elevated eHSP72 suggests that the additional heat stimulation may have amplified eHSP72 levels by activating stress response pathways already induced by exercise.

Immunoglobulins play a crucial role in the body's immune system; IgA is responsible for respiratory and digestive mucosal immunity, while IgG circulates through the bloodstream and mediates systemic immunity against pathogens.^65^^,^^66^ In our study, IgA and IgG levels significantly increased in all three groups. Mohammadnia et al.^67^ has reported an increase in IgA level after 12 weeks of moderate-intensity aerobic exercise, and Pilch et al.^68^ demonstrated that 10 sessions of heat stimulation increased both IgA and IgG levels. Conversely, high-intensity exercise promotes the secretion of stress hormones, such as cortisol and inflammatory cytokines, which may impair immune function for 3–72 h after exercise.^22^^,^^23^

To prevent the decline in immune function in men with obesity and low physical fitness levels, we induced eHSP72 expression with heat stimulation after high-intensity interval training. Previous mechanistic studies have shown that eHSP72 can inhibit the secretion of TNF-α and IL-1β.^69^ In experimental models, eHSP72 has been shown to protect the epithelial cells by inhibiting the NF-κB pathway, which has been associated with reduced inflammatory responses in those experimental systems. Although we did not directly measure these molecular pathways in the present study, previous mechanistic research from cell culture and animal models provides a plausible biological framework for interpreting our findings. Based on previous experimental evidence, eHSP72 has been demonstrated to regulate immune cell function and antibody production through multiple intracellular signaling pathways.^70^^,^^71^ Acting as a danger-associated molecular pattern (DAMP), eHSP72 can bind to receptors such as TLR2, TLR4, and CD91, potentially activating NF-κB, MAPK, and PI3K/Akt cascades.^72^, ^73^, ^74^ In these experimental systems, activation of NF-κB has been associated with transcription of anti-inflammatory cytokines while suppressing pro-inflammatory mediators, whereas MAPK and Akt signaling have been linked to B-cell activation, class switching, and immunoglobulin synthesis.^44^^,^^75^ It is important to note that these pathways were not assessed in the present study; therefore, their role in our observed outcomes remains hypothetical. The observed correlation between increased eHSP72 levels and elevated IgA and IgG concentrations suggests that these molecular mechanisms may contribute to the observed increases in IgA and IgG in our participants.^76^^,^^77^ Moreover, previous research indicates that cross-talk between NF-κB and MAPK signaling may regulate metabolic inflammation, potentially linking reduced adipose tissue dysfunction with enhanced immunity. These proposed mechanisms are based on previous experimental evidence and require direct experimental validation in future studies measuring these specific signaling pathways in human intervention contexts. Thus, the observed associations in our study suggest that eHSP72 may serve as a potential molecular mediator that links cellular stress responses with immunometabolic adaptations induced by combined high-intensity interval training and heat stimulation. However, direct mechanistic evidence would be required to confirm this hypothesis.

In our study, eHSP72 levels increased in all the groups after 12 weeks of training, which suggests that the increased eHSP72 expression was associated with improvements in immunoglobulin levels. These findings indicate that both post-exercise heat stimulation and heat stimulation alone were associated with improved immune function in our participants. However, the observed elevation in eHSP72 cannot be entirely excluded as being attributable to a low-grade inflammatory state in individuals with obesity or to residual effects from the final session measured 48 h later. Therefore, future studies should incorporate assessments of pro-inflammatory markers and multiple post-intervention time points to clarify the underlying mechanisms of these changes. Furthermore, practical considerations such as accessibility, safety, and compliance of heat therapy in diverse populations should also be addressed to enhance the applicability and real-world feasibility of this combined intervention.

Although the 12-week intervention provided valuable findings on the combined effects of high-intensity interval training and heat stimulation, the long-term implications for chronic inflammation and obesity-related immune dysfunction remain to be fully elucidated**.** Repeated exposure to both exercise and heat stimulation over an extended period may induce cumulative adaptations in adipokine regulation, inflammatory signaling, and immune cell function. Consistent with findings from previous mechanistic research, the elevated eHSP72 observed in our study was correlated with immune marker improvements, which may reflect immune modulation through TLR-mediated activation of intracellular pathways such as NF-κB and MAPK, potentially resulting in cytokine regulation and enhanced immunoglobulin production. Collectively, the observed correlations provide a plausible basis for suggesting that elevated eHSP72 expression may be associated with immune adaptations to repeated heat stimulation combined with high-intensity interval training. However, definitive causal relationships would require future mechanistic studies involving pathway inhibition or receptor blockade experiments.

These findings suggest that repeated exposure to high-intensity interval training combined with heat stimulation may induce favorable long-term adaptations in adipokine regulation and immune function. Sustained improvements in adiponectin levels may enhance metabolic flexibility, improve insulin sensitivity, and potentially contribute to improved metabolic health, which is critical for preventing metabolic diseases such as type 2 diabetes and cardiovascular disorders. Likewise, long-term modulation of immunoglobulin levels associated with eHSP72 expression may strengthen immune surveillance and potentially reduce susceptibility to chronic inflammatory conditions in individuals with obesity. Further longitudinal and mechanistic studies are warranted to confirm and extend these preliminary findings.

### 4.1 Limitations

This study has several limitations. First, only male participants with obesity were included, and the relatively small sample size (n = 16 per group) limits generalizability to other populations and may have constrained detection of smaller effects. Second, we did not assess functional immune outcomes, clinical endpoints, or inflammatory cytokines (TNF-α, IL-1β, IL-6). Additionally, potential confounding factors related to heat exposure (hydration status, circadian variation, individual heat tolerance) and unmonitored dietary variations may have influenced outcomes. Third, we examined multiple outcome variables without adjusting for multiple comparisons, increasing Type I error risk. While hypotheses were defined a priori, future confirmatory studies should employ more stringent statistical controls or designate pre-specified primary outcomes. Fourth, we did not perform mechanistic experiments (pathway inhibition, receptor blockade) to establish causal relationships between eHSP72 and immune adaptations. The proposed mechanisms are based on previous experimental evidence, and definitive causal relationships require confirmation through future mechanistic investigations. Finally, although we discussed potential anti-inflammatory mechanisms, we did not measure inflammatory cytokines to confirm whether the intervention affected inflammatory responses, and such interpretations remain speculative.

## 5 Conclusion

In conclusion, this study provides preliminary evidence that the combined intervention of high-intensity interval training and heat stimulation is associated with favorable changes in adipokine regulation, eHSP72 expression, and immunoglobulin levels in men with obesity. Unlike conventional approaches that apply exercise or heat therapy independently, this integrated strategy offers a novel and practical method to enhance both metabolic and immunological adaptations associated with obesity. Moreover, heat stimulation alone was effective in increasing eHSP72 expression and improving immunoglobulin levels, suggesting its potential role in supporting immune function. These findings highlight the novelty and clinical relevance of combining high-intensity interval training with heat stimulation as an innovative therapeutic approach to improve metabolic health and immune regulation in individuals with obesity.

## Informed consent statement

Written informed consent was obtained from all participants after explaining the purpose, procedures, and potential risks of the study. All participants voluntarily agreed to take part, and additional written informed consent was obtained for the publication of this paper.

## CRediT author statement

Dohyun Kim: Conceptualization, Methodology, Formal analysis, Investigation, Writing – Original Draft, Writing – Review & Editing, Supervision, Project administration, Writing – Review & Editing.

Eunsook Kim: Data curation, Investigation, Writing – Review & Editing.

Jinseok Lee: Validation, Writing – Review & Editing.

Sungjin Yoon: Supervision, Project administration, Funding acquisition, Methodology, Resources, Writing – Review & Editing.

## Ethics approval statement

This study was approved by the Institutional Review Board of Korea University (KUIRB-2024-0134-01). All procedures were conducted in accordance with the guidelines of the Institutional Review Board and the Declaration of Helsinki (1964).

## Funding statement

This work was supported by the 10.13039/501100002701Ministry of Education of the Republic of Korea and the National Research Foundation of Korea (NRF-2022S1A5A2A01045974).

## Declaration of competing interest

The authors declare that they have no known competing financial interests or personal relationships that could have appeared to influence the work reported in this paper.
